# The Brazilian version of STarT Back Screening Tool - translation, cross-cultural adaptation and reliability^*^

**DOI:** 10.1590/bjpt-rbf.2014.0028

**Published:** 2014

**Authors:** Bruna Pilz, Rodrigo A. Vasconcelos, Freddy B. Marcondes, Samuel S. Lodovichi, Wilson Mello, Débora B. Grossi

**Affiliations:** 1 Núcleo de Estudos e Pesquisa, Instituto Wilson Mello, Campinas, SP, Brazil; 2 Programa de Pós-graduação em Reabilitação e Desempenho Funcional, Faculdade de Medicina de Ribeirão Preto (FMRP), Universidade de São Paulo (USP), Ribeirão Preto, SP, Brazil; 3 Universidade Estadual de Campinas (UNICAMP), Campinas, SP, Brazil; 4 Centro Universitário da Fundação Educacional de Guaxupé (UNIFEG), Guaxupé, MG, Brazil

**Keywords:** low back pain, questionnaire, STarT Back Screening Tool, rehabilitation, reliability

## Abstract

**Background:**

Psychosocial factors are not routinely identified in physical therapy assessments, although they can influence the prognosis of patients with low back pain. The "STarT Back Screening Tool" (SBST) questionnaire aids in screening such patients for poor prognosis in the primary care setting and classifies them as high, medium, or low risk based on physical and psychosocial factors.

**Objectives:**

This study sought to translate and cross-culturally adapt the SBST to the Brazilian Portuguese language and test the reliability of the Brazilian version.

**Method:**

The first stage of the study consisted of the translation, synthesis, and back-translation of the original version of the STSB, including revision by the Translation Group, pretest of the translated version, and assessment by an expert panel. The pre-final Brazilian version was applied to 2 samples comprising 52 patients with low back pain; these patients were of both genders and older than 18 years of age. To assess the instrument's reliability, an additional sample comprising 50 patients was subjected to 2 interviews, and the results were assessed using the quadratic weighted kappa value. The instrument's internal consistency was assessed using Cronbach's alpha (n=105), and the standard error of measurement was also calculated (n=50).

**Results:**

Translation and back-translation attained consensus, and only item 6 required changes; the reformulated version was applied to an additional sample comprising 52 individuals who did not report any doubts related to this item. The reliability of the SBST-Brazil was 0.79 (95% confidence interval: 0.63-0.95), the internal consistency was 0.74 for the total score and 0.72 for the psychosocial subscale, and the standard error of measurement was 1.9%.

**Conclusion:**

The translated and cross-culturally adapted SBST-Brazil proved to be reliable for screening patients according to their risk of poor prognosis and the presence of psychosocial factors.

## Introduction

Low back pain is a major health problem worldwide, affecting mostly females and individuals aged 40-80 years. Approximately 11.9% of patients exhibit limitations due to low back pain for more than 1 day, and 23.2% of patients show limitations for more than 1 month1. Most patients with acute low back pain (90%) recover within 6 weeks, but symptoms remain in 2 to 7% of patients. These symptoms progress into chronic pain, which accounts for 75-85% of absenteeism in the workplace^2^. In addition, 53% of individuals with chronic low back pain from a specific population exhibit significant psychological disorders^3^. 

The emotional and behavioral impact of this pain favors the development of chronic conditions4-6, and some evidence shows that psychosocial factors, including the patient's perception about the resolution of the symptoms of low back pain and their association with other diseases, difficulty in coping with the disease, lack of confidence, pain catastrophizing, and depressive symptoms, are predictive of dysfunction and interfere with the prognosis of low back pain^7^^-^ 12. Identification in the primary care setting of patients exhibiting psychosocial factors liable to interfere with their prognosis^2^^,^ 7 ^,^ 8 ^,^ 13 contributes to establishing more specific treatments and allows the patient to better understand the consequences of the signs and symptoms of low back pain^13^. These facts notwithstanding, the influence of psychosocial factors is not fully understood and is poorly considered in the planning of treatment. For these reasons, the identification of such factors still poses a challenge^5^^,^ 8. 

Thus, the application of a questionnaire to assess psychosocial factors may enable the stratification of individuals with low back pain and contribute to therapeutic decision-making. 

Recently, Hill et al.^14 ^formulated the "STarT Back Screening Tool" (SBST) questionnaire. Developed in English, the SBST classifies the risk of poor prognosis of low back pain patients with or without radiculopathy influenced by physical and psychosocial factors. The SBST was shown to be able to predict future dysfunction in patients with low back pain in the primary care setting and exhibited acceptable test-retest reliability and internal consistency^15^. 

Several studies have tested the effectiveness of the SBST^12^^,^ 13 ^,^ 15 ^-^ 17. Hill et al.^18^found that patients stratified and treated based on the SBST exhibited better performance on the Rolland-Morris Disability Questionnaire and consequently better quality of life, less use of healthcare services, and lower number of days off work compared to the control group, which was not stratified. 

However, there are few questionnaires available in Brazil to assess the risk of poor prognosis among patients with low back pain influenced by physical and psychosocial factors. For this reason, the aims of the present study were to translate and cross-culturally adapt the SBST to the Brazilian Portuguese language and to analyze its psychometric properties of reliability through assessment of intra-rater reliability, internal consistency, and standard error of measurement to provide a reliable tool for screening individuals with low back pain. Such a tool will afford physical therapists with a differentiated approach and improve their clinical decision-making skills in both the clinical and research settings. 

### Method Description of the SBST questionnaire

The SBST questionnaire is comprised of 9 items. Of these, 4 are related to refererred leg pain, disability, and comorbid shoulder or neck pain, and 5 of the items make up a psychosocial subscale (items 5 to 9) that investigates bothersomeness, pain catastrophizing, fear, anxiety, and depression^12^^,^ 14 ^,^ 15 ^,^ 18. The SBST-Brazil includes the changes introduced and the order of the items formulated by Fritz et al.^12 ^and Hill et al.^18^, which the authors of the original instrument recommended to facilitate patient classification. 

Using the SBST in the study, patients were classified as having a high risk of poor prognosis (high levels of psychosocial prognostic factors were present with or without the physical factors present); medium risk (physical and psychosocial factors were present, but not a high level of psychosocial factors); or low risk (few physical or psychosocial prognostic factors were present)^12^^,^ 18. 

**Figure 1:**
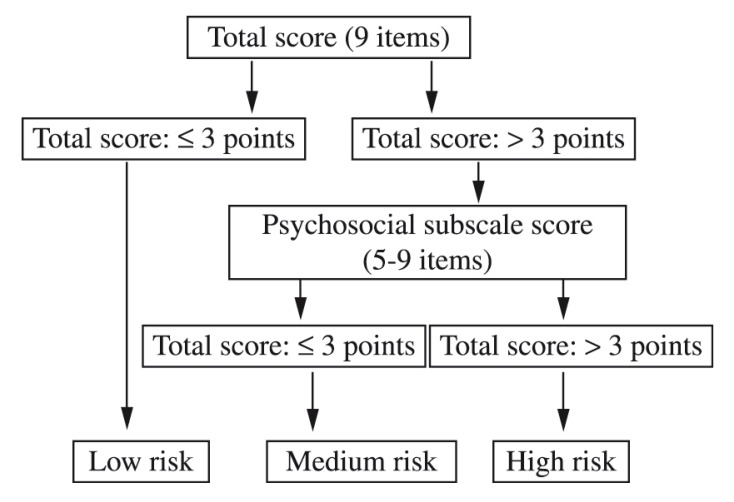
SBST scoring system12,14,18

For the purposes of scoring and classification, respondents were given answer options of "I agree" and "I disagree" for the first 8 items, which were scored 1 and 0 points, respectively. Item 9 had 5 answer options, including "Not at all, Slightly, Moderately, Very much, and Extremely"; the first 3 options were assigned 0 points, and the latter 2 were given 1 point each. Total scores from 0-3 corresponded to a low risk. For total scores greater than 3, classification was based on the psychosocial subscale score (items 5 to 9) as follows: scores ≤3 corresponded to medium risk and scores >3 corresponded to high risk^12^^,^ 14 ^,^ 18. Figure 1 depicts the SBST classification system. 

### Translation and cross-cultural adaptation

Cross-cultural adaptation of the SBST questionnaire was performed using the methods described by Beaton et al.^19^. Authorization for this process was requested from the author of the original version, Dr. Jonathan Hill, Keele University, United Kingdom. The present study was approved by the Research Ethics Committee of the Pontifical Catholic University of Campinas (Pontifícia Universidade Católica de Campinas - PUC-Campinas), Campinas, São Paulo, Brazil, under ruling no. 150.139. 

The modified SBST version^12^^,^ 18 was used for cross-cultural adaptation, which was performed in the following 6 steps: (1) translation, (2) synthesis, (3) back-translation, (4) revision by the Translation Group, (5) pretest, and (6) assessment by an expert panel. 

First, the SBST, which was originally developed in English, was independently translated into Brazilian Portuguese by 2 bilingual public translators (T1 and T2) who were native Portuguese speakers and fluent in English; only one of these translators had knowledge about health subjects. The resulting translations (T1 and T2) were then analyzed together with the original questionnaire by the translators and the investigators (second synthesis step), resulting in version T12. 

In the third step, version T12 was back-translated into English by 2 different bilingual translators (BT1 and BT2) who had no knowledge about the original English version of the questionnaire; these translators were native English speakers residing in Brazil and fluent in Brazilian Portuguese. 

In the fourth step, all 5 versions (original, T1, T2, T12, BT1, and BT2) were revised by the Translation Group, which comprised one physical therapist, 2 orthopedic doctors, and all 4 translators. The Translation Group consolidated all 5 versions to produce a pre-final version of the SBST questionnaire. 

In the fifth step, 2 pretests were performed with the pre-final version to eliminate any item not understood by more than 20% of the sample^20^. In the sixth step, all of the reports were submitted to the Committee for approval, after which they were sent to the author of the original version to approve the final version. 

A convenience sample was recruited at Wilson Mello Institute, Campinas, São Paulo, Brazil. This sample comprised individuals older than 18 years of age with low back pain, independent of the time elapsed since the onset of their back pain and with or without extension of pain to the lower limbs. 

Individuals with severe clinical problems (e.g., cauda equina syndrome, fracture of the lumbar spine, malignancy, and cognitive, neurologic, or rheumatologic disorders), those subjected to lumbar spine surgery within the previous 6 months, pregnant women, and those who could not read or speak Brazilian Portuguese were excluded from the study. All patients who agreed to participate in this stage of the study and in the reliability assessment signed an informed consent form and provided demographic information, which is summarized in Table 1. The questionnaire was self-administered, and once the patients answered the pre-final version, they were each questioned by one of the investigators as to their understanding of each item to formulate suggestions for improvement. 

**Table 1:** Demographic characteristics and SBST-Brazil results of the subjects involved in the study at baseline.

Demographics variables	Pre-final version I (n=52)	Pre-final version II (n=52)	Intra-rater reliability SEM (n=50)	Internal Consistency (n=105)
Age (years), mean (SD)	48.87 (16.1)	50.1 (19.3)	48 (14.5)	47.8 (14.2)
Gender, n (%)				
Male	18 (34%)	24 (46%)	23 (46%)	55 (52%)
Female	34 (66%)	28 (54%)	27 (54%)	50 (48%)
BMI, mean (SD)			25.35 (3.6)	26.3 (4.5)
Education, n (%)				
Primary education level	17 (32.6%)	16 (30.7%)	0 (0%)	26 (24.7%)
High school level	33 (63.4%)	31 (59.6%)	15 (30%)	46 (43.8%)
University level	2 (4%)	5 (9.7%)	35 (70%)	33(31.5%)
NRS, mean (SD)			5.6 (1.9)	5.5 (2.2)
SBST- Brazil, mean (SD)			2.64 (2.16)	4.1 (2.2)
Low risk, n (%)			26 (52%)	53 (50%)
Medium risk, n (%)			17 (34%)	28 (26%)
High risk, n (%)			7 (14%)	24 (24%)

BMI : body mass index

NRS : numeric rating scale

SEM : standard error measurement

SD : standard deviation.

### Psychometric properties

### Reliability

All of the properties corresponding to domain reliability were tested, including intra-rater reliability, internal consistency, and standard error of measurement^21^. Inter-rater reliability was not tested because the STBS is a self-administered questionnaire not subjected to any interference by examiners. 

### Intra-rater reliability

To assess the intra-rater reliability of the SBST-Brazil questionnaire, a different sample comprising 50 patients with unspecific low back pain was subjected to 2 interviews, as recommended by the Consensus-based Standards for the Selection of Health Measurement Instruments (COSMIN)^21^. The patients were recruited by means of convenience sampling at the physical therapy service of the Wilson Mello Institute, Campinas, São Paulo, Brazil. 

The 2 interviews were conducted with an interval of 2 to 7 days, depending on the availability of the patients, and the SBST-Brazil questionnaire and a numerical rating scale (NRS) for pain^22 ^were applied to all subjects. The NRS scale was used only as an exclusion tool. Only stable patients (i.e., those whose NRS scores exhibited variation of 2 points or less between both assessments) were considered for the study, because this value was considered as the minimal clinically important difference (MCID) for patients with chronic low back pain^23^. Patients with variations in the NRS score greater than 2 points and those who missed the second interview were excluded from the study. 

### Internal consistency

A pilot study was conducted in which the SBST-Brazil questionnaire was applied to 105 patients with low back pain to test its internal consistency using Cronbach's alpha. The initial results, showing a value of 0.59 in the total score and 0.51 in the psychosocial scale, were considered unacceptable^21^. Analysis of the characteristics of the sample showed wide heterogeneity relative to the variables educational level (9% had completed primary education only, 20% secondary education, and 71% had completed higher education) and risk stratification based on the SBST (40% were classified as low risk, 43% as medium risk, and 17% as high risk). Thus, a new sample (n=105) more homogeneous in regard to educational level (primary education: 24.7%; secondary education: 43.8%; complete higher education: 31.5%) and risk stratification (low risk: 50%; medium risk: 26%; high risk: 24%) was recruited to test the internal consistency of the SBST-Brazil questionnaire. 

### Standard Error of Measurement (SEM)

The SEM was calculated based on data corresponding to the sample that participated in the first interview for reliability analysis. The SEM does not represent actual changes in the questionnaire results but instead error in measurement^24^. 

### Statistical analysis

Intra-rater reliability was assessed using the quadratic weighted kappa coefficient with the corresponding 95% confidence interval (CI). Following the methods of Sim and Wright^25^, reliability values were classified as poor (≤0), slight (0.01-0.2), fair (0.21-0.40), moderate (0.41-0.60), substantial (0.61-0.80), and almost perfect (0.81-1.0). Values equal to or higher than 0.70 were expected to be found for the reliability of the SBST, according to COSMIN recommendations^21^. Analysis was performed using the software SAS (version 9.2). 

The data relative to internal consistency were tested using Cronbach's alpha, with values within the 0.70-0.95 range considered acceptable^24^. 

SEM was calculated according to the equation SEM=1.96*SD*√(1 - Kappa) with the 95test-retest corresponding 95% CI^26^. Results equal to or lower than 5% were considered very good; 5.1 to 10% as good; 10.1 to 20% as questionable; and above 20.1% as poor^27^.

## Results

The demographic characteristics of the patients involved in the various steps of the study are described in Table 1. 

### Cross-cultural adaptation

Initial cross-cultural adaptation of the SBST questionnaire to Brazilian Portuguese resulted in similar versions, with translations T1 and T2 exhibiting small differences. Table 2 shows how these differences were solved. As a result, the first and second steps of the process were completed, resulting in version T12. 

**Table 2:** SBST- Brazil questionnaire translation process modification.

ITEM	Original Version	T 1	T 2	T 12
3	I have only walked short distances	Evito andar longas distâncias	Eu somente andei curtas distâncias	Eu evito andar longas distâncias
4	I have dressed more slowly than usual	Demora para eu me vestir	Tenho me vestido mais lentamente que o habitual	Tenho me vestido mais devagar
5	It's really not safe for a person with a condition like mine to be physically active	A atividade física é perigosa para as pessoas com a minha doença	Não é realmente seguro para uma pessoa com uma condição como a minha para ser fisicamente ativo	A atividade física não é realmente segura para uma pessoa com um problema como o meu
6	Worrying thoughts have been going through my mind often	Fico preocupado frequentemente	Pensamentos preocupantes têm passando na minha mente	Tenho ficado preocupado por muito tempo

Analysis of the back-translations showed that versions BT1 and BT2 were quite similar and equivalent to the original version of the SBST questionnaire, thus indicating that version T12 was adequate to obtain the pre-final version. 

The first pretest indicated that only item 6 of the questionnaire required changes, as the statement "Tenho ficado preocupado por muito tempo [Worrying thoughts have been going through my mind a lot of the time]" was not understood by more than 20% of the participants^20^. Following revision by the Translation Group and complying with a suggestion made by the author of the SBST, the text of item 6 was changed to "Tenho ficado preocupado por muito tempo por causa da minha dor nas costas [Worrying thoughts have been going through my mind a lot of the time due to the pain in my back]". Following this change, the patients did not report any doubts on the second pretest, and thus the final Brazilian Portuguese version of the SBST was established, which is presented in Appendix 1. 

### Reliability

The intra-rater reliability was considered to be substantial^25 ^according to the reference values selected, as the result was greater than 0.70, which is the established minimum^21^. 

The internal consistency values of the SBST-Brazil questionnaire were also acceptable (total score: 0.74; psychosocial subscale: 0.72), and the SEM was rated very good. These results are described in Table 3. 

**Table 3:** Intra-rater reliability (n=50), internal consistency (n=105) and SEM (n=50) results for the SBST-Brazil questionnaire.

	Classification Low/ Medium/High risk (95% CI)	Total Score	Psychosocial Subscale Score
**Intra-rater reliability** (Quadratic weighted kappa)	0.79 (0.63-0.95)		
Internal consistency		0.74	0.72
SEM (%)		1.9	

SEM : Standard error measurement.

## Discussion

Reliable application of foreign questionnaires to the Brazilian population demands their systematic and judicious cross-cultural adaptation to the Brazilian Portuguese language. Cross-cultural adaptation of specific questionnaires is not simple, as not only language-related but also cultural differences between countries should be taken into consideration for the validity and reliability of instruments to be preserved^19^^,^ 26. For these reasons, the cross-cultural adaptation of the SBST questionnaire was performed with utmost care relative to the semantic, idiomatic, and conceptual equivalence, while preserving the original concepts^28^. Only item 6 posed doubts to the patients, and changes in its text were suggested by the original author of the SBST to conserve its intention. As a result, the new text was approved by the expert panel, and retest using a different sample showed that this item no longer posed doubts regarding its meaning. 

The SBST-Brazil is the first Brazilian questionnaire designed to screen and classify patients with low back pain as to their risk of poor prognosis relative to physical therapy due to psychosocial factors. Although the original version of the SBST was translated to other languages, including Spanish, French, Danish, Arabic, Dutch, German, Italian, Polish, Norwegian, Mandarin, Japanese, Swedish, Turkish, Urdu, Welsh, and Yoruba^29^, no adaptation to Brazilian Portuguese was available. 

In our analysis, the quadratic weighted kappa value was 0.79 (95% CI: 0.63-0.95), which indicates that the intra-rater reliability of the SBST-Brazil was acceptable relative to the classification result and close to the value of the original version^14^, which are 0.73 (95% CI: 0.57-0.84) for the total score and 0.76 (95% CI: 0.52-0.89) for the psychosocial subscale. The internal consistency results were greater than 0.70 (total score: 0.74; psychosocial subscale: 0.72) and these values are similar to those corresponding to the SBST original^14 ^(total score: 0.79; psychosocial subscale: 0.74), French^30 ^(psychosocial subscale: 0.74), and Iranian^31 ^(total score: 0.82; psychosocial subscale: 0.79) versions, all of which are recommended for use in clinical and research settings. To date, only internal consistency values for the latter 2 versions have been reported, and the samples used in those versions did not comprise as broad a scope of clinical conditions as that in the present study. Indeed, the Brazilian version of the SBST achieved acceptable values using a sample comprising patients with a broad scope of clinical conditions, including unspecific low back pain and postoperative low back pain, with or without arthrodesis, spondylolisthesis, foraminal stenosis, and degenerative central canal stenosis, and thus was representative of the real-world physical therapy setting. Despite the wide variety of clinical conditions, the sample used in the present study exhibited homogeneous distribution as to the patients' educational level and risk stratification. In comparison, the sample used in the pilot study previously conducted to test the internal consistency of the SBST-Brazil included a low percentage of patients with low educational levels and those classified as high risk, and the resulting internal consistency was less than 0.70 (total score: 0.59; psychosocial subscale: 0.51). However, this sample achieved greater representativeness after the variables risk level and educational level were more homogeneously distributed. As a result, the final SBST-Brazil demonstrated satisfactory internal consistency for use in patients with various clinical low back pain conditions. 

In regard to the internal consistency of the SBST-Brazil, it is worth noting that this instrument focuses on the assessment of psychosocial aspects related to coping that can be strongly influenced by the psychosocial profile of the sample. Therefore, future studies will also be able to establish whether the internal consistency values of the SBST-Brazil remain acceptable in individuals corresponding to the same clinical, diagnostic, or sociocultural strata. 

The SEM calculated for the SBST-Brazil was classified as very good^27^. This result also indicates that the actual score of any individual may vary 1.9% above or below the score attained in the applied questionnaire, which is not indicative of any real change in the patient's clinical condition but merely reflects an error in measurement. 

The reliability domain exhibited satisfactory results that were quite similar to those corresponding to the original version of the questionnaire, indicating that the SBST-Brazil is reliable for application to the Brazilian population. 

Use of the SBST can lead to significant differences in the standard treatment provided to different groups of individuals with low back pain in the primary care setting^18^. Patients classified as high risk using this instrument exhibit unfavorable prognosis due to the presence of psychosocial factors, and may not have access to specif treatment what includes physical and psychosocial components based on cognitive and behavioral principles, and thus could not exhibit satisfactory outcomes. Although the prognosis of patients classified as medium risk is less unfavorable compared to those classified as high risk, these subjects also require physical therapy, mainly because of their physical symptoms. The prognosis of individuals classified as low risk is good, and they may benefit from advice and explanation about their symptoms, reassurance, education about their daily and work activities, with no need for physical therapy on a steady basis. The abovementioned features show that the SBST allows physical therapists to define more accurately the best approach to treatment for each individual patient. 

Upon comparing subjective decision-making by clinical experts to the SBST's allocation to risk subgroups, Hill et al.^17 ^found that the agreement between the group assessed by clinicians and the group assessed using the SBST was poor. In addition, the results of the latter group were better than those of the former, which is indicative of the difficulties clinicians encounterin identifying individuals at high risk of poor prognosis. The SBST aids in the identification of individuals who require special care and thus represents an important adjuvant to clinical assessment. 

Nevertheless, the SBST has some limitations, including the failure to identify psychosocial problems in individuals without pain complaints and the inability to specify the patient's preferences, expectations, and past treatments^17^. The usefulness of the SBST for screening patients with low back pain notwithstanding, other questionnaires should be used during clinical follow up, such as the Fear-Avoidance Beliefs Questionnaire (FABQ) or the shortened version of the Tampa Scale for Kinesiophobia (TKS-11)^15^. 

The SBST can contribute to the initial screening of individuals with low back pain to improve their treatment, as well as to the performance of clinical studies of individuals with low back pain. In addition, based on the present study, other psychometric properties of the SBST may also be assessed. 

## Conclusion

The translation and cross-cultural adaptation of the SBST to the Brazilian Portuguese language was performed in a satisfactory manner. The resulting SBST-Brazil version proved to be reliable for use in Brazil, thus contributing to the treatment of individuals with low back pain in the primary care setting by screening them for a risk of poor prognosis and taking psychosocial factors into account. 

## Appendix 1 STarT Back Screening Tool- Brasil (SBST-Brasil).


